# Peripheral Oxidation-Inflammation and Immunosenescence in Triple-Transgenic Mice for Alzheimer’s Disease (3xTg-AD) at Early Neuropathological Stages of Disease and Decrease of Immune Impairment by Voluntary Exercise

**DOI:** 10.3390/biom16030475

**Published:** 2026-03-22

**Authors:** Mónica De la Fuente, Antonio Garrido, Carmen Vida, Rashed Manassra, Lydia Gimenez-Llort

**Affiliations:** 1 Department of Genetics, Physiology and Microbiology (Animal Physiology Unit), Faculty of Biological Sciences, Complutense University of Madrid, 28040 Madrid, Spain; mondelaf@bio.ucm.es (M.D.l.F.); mvida@ucm.es (C.V.); rasshed.manassra@gmail.com (R.M.); 2 Institute of Investigation Hospital 12 Octubre (i+12), 28041 Madrid, Spain; 3 Nanocaging Research Group, Department of Biosciences, School of Biomedical and Health Sciences, Universidad Europea de Madrid, Villaviciosa de Odón, 28670 Madrid, Spain; 4 Institute of Neuroscience, Universitat Autònoma de Barcelona, Campus Bellaterra, 08193 Barcelona, Spain; lidia.gimenez@uab.cat; 5 Department of Psychiatry and Forensic Medicine, Medicine School, Universitat Autònoma de Barcelona, Campus Bellaterra, 08193 Barcelona, Spain

**Keywords:** oxidation-inflammation, immunosenescence, triple-transgenic Alzheimer disease (3xTg-AD) mouse model, early neuropathological state, physical exercise

## Abstract

Inflammatory-oxidative stress generated by immune cells plays an important role in aging and in age-related neurodegenerative disorders such as Alzheimer’s disease (AD). Triple-transgenic mice for AD (3xTg-AD) are a suitable model for mimicking this disease in an age-dependent manner. We previously showed that peritoneal leukocyte functions and their redox-inflammatory state are altered early in female 3xTg-AD mice, which exhibit premature aging compared to non-transgenic (NTg) animals. However, their characteristics at 9 months of age, when they present an early neuropathological state, and the sex differences are not known. Here, we analyzed several spleen and thymus leukocyte functions (chemotaxis, natural killer activity, and lymphoproliferation in response to mitogens), pro-inflammatory (IL-1B, TNF-alpha) and anti-inflammatory (IL-10) released cytokine concentrations, and redox parameters (glutathione concentrations and glutathione peroxidase, glutathione reductase, and xanthine oxidase activities) in male and female 3xTg-AD mice compared to age-matched controls. We also analyzed the effects of voluntary physical exercise on immune functions. Our results show that 9-month-old male and female 3xTg-AD mice have worse immune functions, redox state, and inflammation than NTg counterparts. Physical exercise improves immune function. Thus, accelerated aging reflected by peripheral immunosenescence and oxidation-inflammation in 3xTg-AD mice precedes hallmark neuropathology, and exercise can slow down AD progression.

## 1. Introduction

The aging process, characterized by the progressive and general decline of physiological functions and the decreased ability to adapt to changes, i.e., a deterioration of homeostasis, is associated, consequently, with increased risk of morbidity. The oxidation-inflammation theory of aging proposes that this process results from chronic oxidative and inflammatory stress (imbalance between oxidant-proinflammatory compounds and antioxidant/anti-inflammatory defenses, in favor of the former), affecting all cells, especially those of the homeostatic systems such as the nervous, endocrine and immune systems, as well as the communication between them. Furthermore, the immune system and its age-related changes, known as immunosenescence, can modulate the aging rate of each individual [1,2]. Thus, oxidation and inflammation—two processes that always occur together [3]—underlie the aging process and age-related diseases.

Alzheimer’s disease (AD) is the most prevalent neurodegenerative disorder associated with aging, and it is characterized at the histopathological level by the presence of intracellular neurofibrillary tangles (NFTs) and numerous extracellular senile (neuritic) plaques (SPs), leading to synaptic loss. SPs are mainly composed of amyloid β-peptide (Aβ), and NFTs are aggregates of the hyperphosphorylated microtubule-associated protein tau [4]. Growing evidence suggests an important role for inflammatory and oxidative stress in the pathogenesis and progression of AD [5,6,7,8,9], and even Aβ itself has been shown to act as an oxidant and proinflammatory agent [10].

Although AD is primarily recognized as a brain disease, its significant impact on peripheral tissues beyond the central nervous system (CNS) has been suggested. In fact, the brain-peripheral communication hypothesis is supported by the wide range of peripheral abnormalities observed in AD patients [11]. Moreover, it is known that the bidirectional connection between the nervous and immune systems, essential in maintaining homeostasis [1,12], is altered with aging and with AD [1,13,14]. Thus, peripheral immune cells can infiltrate the brain, especially when the permeabilization of the blood–brain barrier (BBB) increases [15], modulating the hallmark features of AD, which include amyloid plaque accumulation, tau pathology and neuroinflammation [16] as well as other aspects of this disease [17], contributing to the pathogenesis of AD [18,19]. Thus, peripheral oxidation-inflammation and immune alterations can exacerbate neurodegeneration in AD, and even more, there is evidence suggesting that peripheral immune alterations may precede or occur alongside brain changes in AD [20]. However, despite considerable progress in AD research, that subject has yet to be clearly elucidated, and, to this end, the use of preclinical models is crucial for understanding these mechanisms in AD pathogenesis and progression. Although no single model fully exhibits the complexity of AD, they are useful in this context [21]. Triple-transgenic Alzheimer’s Disease (3xTg-AD) mice harbor *PS1M146V*, *APPSwe* and *tauP301L* transgenes, a mouse model developed in 2003 by LaFerla’s laboratory [22,23]. These animals mimic, in an age-dependent manner, both amyloid and tau AD signatures, developing plaques and tangles and exhibiting synaptic dysfunction, which includes LTP deficits as well as cognition impairments and behavioral symptoms of dementia, characteristics that make these mice a valid model for studying AD [22,24,25,26]. In 3xTg-AD mice, intracellular Aβ accumulation begins at 2 months of age, followed by extracellular Aβ plaque formation at 6 months of age, which becomes widespread by 12 months together with neuroinflammation. The Tau pathology occurs later, starting around 12 months and becoming consistently detectable by 15 months [25,26,27,28].

Previously, we reported that the crosstalk between the homeostatic systems, which constitute a neuroimmunoendocrine network that ensures homeostatic maintenance, is early disrupted in 3xTg-AD mice, which is associated with a short lifespan of these mice. Furthermore, peripheral immune functions were proposed as markers of general neuroimmunoendocrine system-related conditions [29,30,31,32]. In this model of mice, we reported the functions, inflammatory and redox state of peritoneal leukocytes from 2-, 4-, 6-, 12- and 15- month-old female 3xTg-AD mice and compared them to those in non-transgenic (NTg) control mice. The results showed premature immunosenescence and oxidative-inflammatory stress in 3xTg-AD mice at an early age (2 and 4 months of age), prior to the onset of AD, but in general, these significant differences in immune functions between 3xTg AD and NTg females in early ages disappear at 12 months, whereas these were maintained in oxidative stress parameters [31,32,33]. Among peripheral immune organs, the spleen has been considered a key organ of the neuroimmune system, and it is related to brain function and health by immune modulation [34]. Moreover, neuroinflammation in AD is associated with changes in both brain and spleen immune cell populations [35,36].

In addition, it is known that the sex-dependent impairment of the immune system functions and oxidative-inflammatory stress occurs with aging [37,38]. These sex differences were also reported in the neuroimmunoendocrine system in 3xTg-AD at 15-month-old, when AD pathophysiology is fully established, compared to old NTg animals, and those were more significant in males, contributing to their increased mortality [39]. In this context, sex differences in the peripheral immune functions and the oxidative-inflammatory state of leukocytes in 9-month-old 3xTg-AD mice, when diffuse plaques of Aβ appear, and animals present an early neuropathological state of disease [26,28], have not yet been studied.

Since aging and age-related diseases, such as AD, are based on oxidative-inflammatory stresses, which affect homeostatic systems, and especially immune system functions, useful extrinsic factors such as certain lifestyle strategies capable of increasing antioxidant and anti-inflammatory defenses as well as immune capacity [2,40] may delay both aging rate and AD progression [41]. Accordingly, one possible intervention may be physical exercise. There is evidence showing that physical exercise has many beneficial physiological effects and is associated with better immune capacity and health [40,42,43,44,45]. However, physical exercise produces different results on immunity depending on the type, intensity, and frequency, as well as the immune function studied and the subject’s state. Intense physical exercise tends to produce adverse changes and may lead to immunosuppression, whereas moderate training exercise improves immune functions in humans and experimental animals [40,44]. Moreover, regular exercise training decreases oxidative stress and improves resistance to oxidative damage due to the downregulation of oxidant release because of the adaptation of antioxidant defenses, which increases their amounts and activities [40,46,47,48]. This shows that exercise is a preventive tool, especially delaying the onset of immunosenescence, against accelerated aging and neurodegeneration, such as that in AD, associated with oxidative challenge [49,50,51,52]. In 3xTg-AD, exercise triggers positive effects on bone density, like those produced by resveratrol ingestion [53], and it has also been reported to mitigate behavioral deficits associated with disease and regulate neuroinflammation [54,55]. However, the effect of voluntary exercise on immune functions in these 3xTg-AD mice has not been studied.

For all the indicated, the aim of this study was to examine several immune functions, inflammatory and anti-inflammatory cytokine release, and redox parameters in leukocytes from the spleen and thymus of 9-month-old male and female 3xTg-AD mice. This age, in which these animals exhibit an early neuropathological state of disease, has been scarcely studied in this context. Additionally, the positive effects of voluntary physical exercise on immune functions were considered.

## 2. Materials and Methods

### 2.1. Animals

Sixty-four triple-transgenic mice for Alzheimer’s disease (3xTg-AD), carrying the PS1M146V, APPSwe, and tauP301L mutations, were included in the study. All animals originated from the C57BL/129Sv background strain. Genotypes were verified by PCR using DNA extracted from tail samples. At six months of age, 32 mice (male and female, both 3xTg-AD and non-transgenic controls) were placed in standard housing, with eight animals per cage. The remaining 32 mice were housed in cages equipped with a running wheel (Activity Wheel Cage System for mice, Techniplast, Buguggiate, Italy) to allow continuous voluntary exercise. Group housing was maintained throughout the experiment to prevent stress associated with isolation. Visual monitoring indicated that all animals were engaged with the wheel, although individual running activity could not be quantified. After three months of housing under these conditions—when the mice reached nine months of age—all animals were euthanized by decapitation. The spleen and thymus were removed immediately under sterile conditions and kept at 4 °C in Hank’s balanced saline solution until leukocyte isolation. Half of each spleen was stored at –80 °C for later assessment of antioxidant defenses. Animals were kept at 22 ± 2 °C in a sterile, negative-pressure environment (Flufrance, Cachan, France) under a reversed 12:12 h light/dark cycle, with lights switched on at 20:00 h. Water and standard Sander Mus pellets (A04 diet, Panlab L.S., Barcelona, Spain) were provided ad libitum, following the nutritional recommendations of the American Institute of Nutrition for laboratory rodents. All procedures were approved by the Institutional Animal Care Committee (PROEX 224.0/21) and complied with European Directive ECC/566/2015 and the ARRIVE guidelines.

### 2.2. Isolation of Leukocytes from Spleen and Thymus

Spleen and thymus tissues were trimmed to remove surrounding fat, cut into small fragments, and gently passed through a mesh filter (Sigma, St. Louis, MO, USA) using phosphate-buffered saline (PBS) as the medium. Spleen cell suspensions were then layered onto a Ficoll-Hypaque gradient (Sigma-Aldrich; density 1.070 g/mL) and centrifuged, after which the leukocyte-rich interface was collected. Leukocytes obtained from both organs were washed with PBS, counted, and adjusted to a final concentration of 1 × 10^6^ cells/mL. Cell viability was assessed before and after each procedure using the trypan blue exclusion method and consistently exceeded 95%. All cell incubations were carried out at 37 °C in a humidified environment containing 5% CO_2_.

### 2.3. Immune Function Parameters

The following immune function parameters were studied in spleen and thymus leukocytes.

#### 2.3.1. Chemotaxis Capacity

The chemotactic response of lymphocytes from spleen and thymus was assessed following a previously established procedure [56]. The assay uses chambers composed of two compartments separated by a 3 µm pore membrane (Millipore Ibérica, Madrid, Spain). A leukocyte suspension (0.3 mL at 1 × 10^6^ cells/mL) was placed in the upper compartment, while the lower compartment contained the chemoattractant FMLP (formyl-Met-Leu-Phe; Sigma-Aldrich, St. Louis, MO, USA) at a concentration of 10^−8^ M. The chambers were incubated for 3 h at 37 °C in a humidified atmosphere with 5% CO_2_. After incubation, the membranes were fixed and stained, and the Chemotaxis Index (CI) was calculated by counting the number of lymphocytes present in four 5 mm fields on the lower surface of the filter under a light microscope at 100× magnification.

#### 2.3.2. Natural Killer (NK) Activity

NK activity in lymphocyte suspensions from the spleen and thymus was evaluated using an enzymatic colorimetric assay designed to quantify the cytolysis of target cells (Cytotox 96™, Promega, Madison, WI, USA; Cytotox 96™, Promega, Boehringer Ingelheim, Germany). This method measures lactate dehydrogenase (LDH) released into the medium through the reduction of a tetrazolium salt [56]. Yac-1 cells, a murine tumor line, served as target cells and were plated in U-bottom 96-well plates (Nunc, Roskilde, Denmark) at 10^4^ cells per well in phenol-red–free RPMI-1640. Effector cells (leukocytes from spleen and thymus) were added at 10^5^ cells per well, establishing an effector-to-target ratio of 10:1. All conditions were tested in triplicate. After centrifugation at 250 *g* for 4 min to promote contact between cells, plates were incubated for 4 h. LDH activity in 50 μL of supernatant from each well was then quantified by adding the enzymatic substrate and measuring absorbance at 490 nm. Three control conditions were included: spontaneous LDH release from target cells, maximum LDH release from target cells, and spontaneous release from effector cells. Cytotoxicity was expressed as the percentage of target-cell lysis, calculated using the corresponding formula:

% lysis = ((E − ES − TS)/(M − ES − TS)) × 100

The variables in the formula were defined as follows: E referred to the average absorbance measured when effector cells were present; ES was the average absorbance of effector cells cultured on their own; TS represented the average absorbance of target cells maintained only with medium; and M corresponded to the average maximum absorbance obtained after exposing target cells to the lysis solution.

#### 2.3.3. Lymphoproliferation Assay

Lymphocyte proliferation from spleen and thymus in response to the mitogens ConcanavalinA (ConA) and lipopolysaccharide (LPS) was evaluated using a previously established procedure [56]. Lymphocyte suspensions were adjusted to 1 × 10^6^ cells/mL in complete medium (RPMI-1640 supplemented with 1% gentamicin and 10% fetal calf serum), and 200 μL of this suspension was distributed into flat-bottom 96-well plates (Nunc, Roskilde, Denmark). Stimulated wells received 20 μL of LPS (1 μg/mL, Sigma) or ConA (1 μg/mL), while control wells received 20 μL of complete medium. All conditions were tested in triplicate. Cultures were maintained for 48 h at 37 °C in a humidified 5% CO_2_ atmosphere. After this incubation period, 100 μL of supernatant was collected for cytokine analysis, and 0.5 μCi of [^3^H]-thymidine (ICN, Costa Mesa, CA, USA) was added to each well. Twenty-four hours later, cells were harvested with a semi-automatic microharvester, and thymidine incorporation was quantified using a beta counter (LKB, Uppsala, Sweden). Results were expressed as counts per minute (cpm), and proliferative responses were presented as a percentage relative to the cpm values of the corresponding unstimulated control wells, which were set to 100%.

#### 2.3.4. Cytokine Concentration Measurements

Cytokine levels for interleukin-2 (IL-2), interleukin-10 (IL-10), interleukin-1B (IL-1B) and tumor necrosis factor-alpha (TNF-alpha) were quantified in the supernatants of spleen- and thymus-derived leukocyte cultures after 48 h of stimulation with ConA (1 µg/mL) or LPS (1 µg/mL). A mouse-specific immunoassay (Millipore MILLIPLEX^®^ MAP Mouse Cytokine/Chemokine kit, Darmstadt, Germany) was used to simultaneously detect and measure these cytokines, with each sample analyzed in duplicate. The assay’s sensitivity threshold for IL-2, IL-10, IL-1B and TNF-alpha was 3.2 pg/mL, and cytokine concentrations were reported in pg/mL.

### 2.4. Redox Parameters

Only a limited set of oxidative-stress markers could be assessed because the remaining spleen and thymus leukocyte samples were scarce. As a result, only reduced glutathione (GSH) levels were measured as an antioxidant indicator and xanthine oxidase (XO) activity as an oxidant parameter. In addition, the portion of each spleen that had been stored at −80 °C allowed for the determination of glutathione peroxidase (GPx) and glutathione reductase (GR) activities.

#### 2.4.1. Reduced Glutathione (GSH) Concentrations

GSH levels were measured spectrophotometrically in both spleen leukocytes and whole-spleen tissue following a previously established protocol [57], with minor adjustments. Spleen samples were homogenized in a cold room (4 °C) using 5% trichloroacetic acid (TCA) and 0.01 N HCl. For spleen leukocytes, 1 × 10^6^ cells were resuspended in the same TCA/HCl solution under the same temperature conditions. All samples were kept on ice throughout the procedure. They were then centrifuged at 3200 *g* for 5 min at 4 °C, and aliquots of the resulting supernatants were analyzed in a spectrophotometer using a reaction mixture containing DTNB (6 mM), β-NADPH (0.3 mM), and glutathione reductase (10 U/mL). The reaction was monitored for 240 s, and measurements were taken in optical glass microcuvettes containing the prepared reaction mixtures.

(1) Blanc: 500 μL NADPH, 70 μL DTNB, 70 μL sample and 70 μL of TCA solution.
(2) Reaction with sample to measure total GSH: 500 μL NADPH, 70 μL DTNB, 70 μL sample and 70 μL GR.
(3) Reaction without sample: 500 μL NADPH, 70 μL DTNB, 70 μL of TCA solution and 70 μL GR.

Standard curves were generated using a glutathione stock solution (Sigma). For this purpose, 16 mg of GSH were dissolved in 250 mL of 5% TCA with 0.01 N HCl, and the resulting standards were analyzed under the same conditions as the experimental samples. Final GSH values were expressed as nmol per mg of tissue, calculated using the corresponding equation:

nmol GSH/mg tissue or nmol GSH/10^6^ cells = [(ΔDO/min) + 0.0021/0.3951] × (1/X)

In this equation, ΔDO/min with sample represents the change in optical density per minute when the sample is present, while ΔDO/min without sample corresponds to the reaction rate measured in the absence of the sample. The term X refers to the product of the sample volume used in the microcuvette (0.07 mL) and the sample concentration (expressed as mg of tissue per mL). For spleen leukocytes, the final values were expressed as nmol of GSH per mg of protein.

#### 2.4.2. Glutathione Peroxidase (GPx) Activity

GPx activity in spleen tissue was determined spectrophotometrically following an established protocol [58] with slight modifications [57]. The decline in absorbance at 340 nm was recorded every 40 s over a 5 min period. Cumene hydroperoxide (Sigma-Aldrich) served as the substrate to oxidize glutathione, while β-NADPH and glutathione reductase (GR) (Sigma-Aldrich) were included to regenerate reduced glutathione. The enzymatic reaction was tracked by monitoring the progressive decrease in absorbance at 340 nm. Spleen samples were homogenized in 50 mM phosphate buffer in a cold room (4 °C) and kept on ice throughout the procedure. They were then centrifuged at 3200 *g* for 20 min at 4 °C, and aliquots of the resulting supernatants were analyzed in a spectrophotometer. GPx activity was calculated using the corresponding formula and expressed as international milliunits of enzyme activity per milligram of tissue (mU·I. GPx/mg tissue):

mU.I. GPx/mg tissue = [(ΔDO/min) × F/Є] × (1/X)

DO/min: ΔDO/min catalyzed reaction − ΔDO/min non-catalyzed reaction

Є: molar absorption coefficient of the NADPH at 340 nm (6.22 × 10^−3^ nM^−1^ cm^−1^).

F: dilution factor of the sample in the microcuvette (700/25 = 28).

X = Volume of the sample in the microcuvette (0.025 mL) × sample concentration (mg tissue/mL)

#### 2.4.3. Glutathione Reductase (GR) Activity

Glutathione reductase (GR) activity in the spleen was quantified using a spectrophotometric method previously established in the literature [59]. Spleen tissue was homogenized at 4 °C in a phosphate buffer (50 mM) containing EDTA (6.3 mM), with the solution kept continuously deoxygenated. The homogenates were then centrifuged at 3200 *g* for 20 min at 4 °C. Supernatants from this step were used for spectrophotometric analysis with NADPH (6 mM) and GSSG (80 mM), both stored in light-protected containers. Measurements were carried out in optical glass microcuvettes containing the reaction mixture. GR activity was determined using the appropriate calculation and expressed as milliunits of enzyme activity per milligram of tissue (mU GR/mg tissue).

mU GR/mg tissue = [(ΔDO/min) × F/Є] × (1/X)

DO/min: ΔDO/min catalyzed reaction − ΔDO/min non-catalyzed reaction

Є: molar absorption coefficient of the NADPH at 340 nm (6.22 × 10^−3^ nM^−1^ cm^−1^).

F: dilution factor of the sample in the cuvet (700/50 = 14).

X: Volume of the sample in the cuvet (0.05 mL) × sample concentration (mg tissue/mL).

#### 2.4.4. Xanthine Oxidase Activity

Xanthine oxidase (XO) activity in spleen leukocytes and spleen tissue was determined using a commercial assay kit (Amplex Red Xanthine/Xanthine Oxidase Assay Kit, A-22182, Molecular Probes, Eugene, OR, USA). Spleen tissues and isolated cells were homogenized in a potassium phosphate buffer containing 50 mM EDTA, 0.1 M phosphate, and 0.5 mM dithiothreitol (DTT). After homogenization, the samples were centrifuged, and the resulting supernatants were used to initiate the enzymatic reaction with the Amplex Red working solution. Fluorescence was recorded at 530 nm for excitation and 595 nm for emission. XO activity was expressed as international milliunits (mU) per milligram of tissue (mU XO/mg tissue) or per milligram of protein for spleen leukocyte samples.

### 2.5. Protein Quantification

Protein levels were determined using the same supernatants prepared for the assessment of redox markers. Quantification was carried out with the bicinchoninic acid (BCA) method, employing the corresponding BCA kit from Sigma-Aldrich. In this assay, proteins in the sample reduce Cu^2+^ to Cu^+^, and the resulting Cu^+^ ions form a colored complex with bicinchoninic acid. The intensity of this complex is then measured spectrophotometrically at 562 nm. Final protein concentrations were reported as milligrams of protein per milliliter (mg/mL).

### 2.6. Statistical Analyses

SPSS 15.0 (SPSS, Chicago, IL, USA) was used to perform all statistical analyses. Results are presented as mean ± standard deviation (SD). The normal distribution of the data and the equality of variances were evaluated using the Kolmogorov–Smirnov and Levene tests, respectively. Group differences were examined with a two-way ANOVA, considering sex and genotype as factors. For variables related to physical exercise, a three-way ANOVA was applied, incorporating sex, genotype, and exercise as factors. When significant effects were detected, Tukey’s test was used for post hoc comparisons. A *p*-value below 0.05 was regarded as statistically significant.

## 3. Results

**Figure 1:**
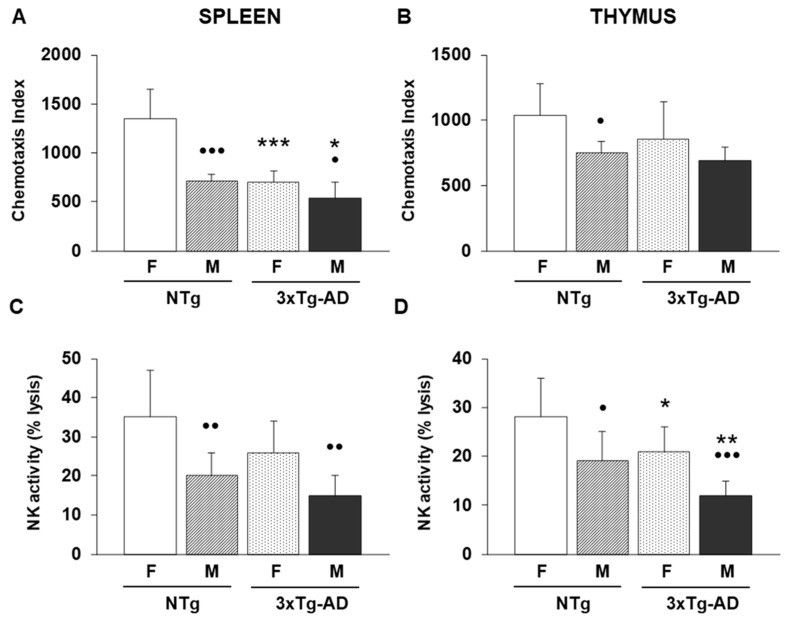
Innate immunity parameters were evaluated in spleen and thymus leukocytes from female and male non-transgenic (NTg) and triple-transgenic Alzheimer’s Disease (3xTg-AD) animals. Data represent the mean ± SD of 8 mice per group. (**A**) Chemotaxis index evaluated in spleen and (**B**) thymus leukocytes. (**C**) Natural Killer (NK) activity, represented as a percentage (%) of lysis, was analyzed in spleen and (**D**) thymus leukocytes. * *p* < 0.05, ** *p* < 0.01, *** *p* < 0.001 with respect to non-transgenic control; • *p* < 0.05, •• *p* < 0.01 and ••• *p* < 0.001 with respect to the values obtained in females of the same genetic condition.

**Figure 2:**
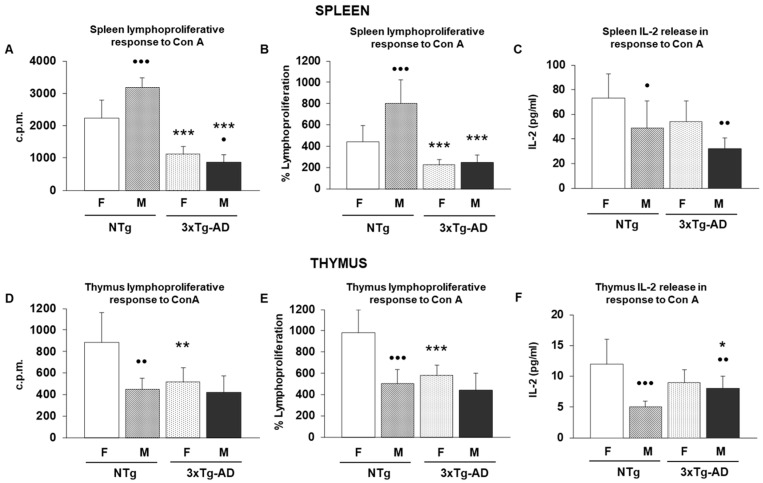
Acquired immunity parameters evaluated in spleen and thymus leukocytes from female and male non-transgenic (NTg) and triple-transgenic Alzheimer’s Disease (3xTg-AD) animals. Data represent the mean ± SD of 8 mice per group. (**A**) Lymphoproliferative response to ConcanavalinA (ConA) represented as counts per minute (c.p.m.) and (**B**) percentage of stimulation (%). (**C**) IL-2 release (pg/mL) in spleen supernatant cultures stimulated with ConA. (**D**) Thymus lymphoproliferative response to ConcanavalinA (ConA) represented as counts per minute (c.p.m.) and (**E**) percentage of stimulation (%). (**F**) IL-2 release (pg/mL) in thymus supernatant cultures stimulated with ConA. * *p* < 0.05, ** *p* < 0.01 and *** *p* < 0.001 with respect to non-transgenic control; • *p* < 0.05, •• *p* < 0.01 and ••• *p* < 0.001 with respect to the values obtained in females of the same genetic condition.

To determine whether, at 9 months of age, triple-transgenic Alzheimer’s disease animals (3xTg-AD), both females and males, exhibit an impairment of the immune system, we analyzed several immune functions related to innate and acquired immunity in their spleen and thymus leukocytes, and also in their non-transgenic counterparts. These results are illustrated in Figure 1 (innate immunity), Figure 2 (acquired immunity) and Table 1 (acquired immunity).

In the case of innate immunity and specifically for the chemotaxis index evaluated in spleen leukocytes (Figure 1A), data showed robust effects of both sex and genotype factors. A two-way ANOVA revealed significant effects of sex (F(1.28) = 39.6, *p* < 0.001) and genotype (F(1.28) = 42, *p* < 0.001), as well as a significant sex × genotype interaction (F(1.28) = 14.1, *p* < 0.001). Female and male 3xTg-AD exhibited a lower value of this immune function compared to female and male non-transgenic (NTg) counterparts (*p* < 0.001 and *p* < 0.05, respectively). In contrast, thymus chemotaxis displayed a significant effect of sex (F(1.28) = 10,2, *p* < 0.001), while genotype factor (F(1.28) = 3, *p* < 0.09) and the interaction (F(1.28) = 0,84, *p* < 0.37) were not significant. Post hoc analyses indicated that although the trend previously described for spleen was observed (Figure 1B), no statistical differences were obtained between 3xTg-AD and NTg animals. NK cell activity in the spleen was also influenced by both factors. Significant main effects were observed for sex (F(1.28) = 20.1, *p* < 0.001) and genotype (F(1.28) = 5.8, *p* < 0.05), whereas the interaction was not significant (F(1.28) = 0.48, *p* = 0.49). No statistical differences between 3xTg-AD and NTg animals were also exhibited in the case of post hoc studies for NK activity analyzed in spleen leukocytes (Figure 1C). Thymus NK activity showed significant effects of sex (F(1.28) = 19.3, *p* < 0.001) and genotype (F(1.28) = 11.7, *p* < 0.01), with no evidence of interaction between the factors (F(1.28) = 0.0, *p* = 1.00). Female and male 3xTg-AD groups had lower percentages of this immune function compared to their respective controls (female and male NTg mice (*p* < 0.05 and *p* < 0.01, respectively) (Figure 1D).

Regarding acquired immunity, mitogenic responses to ConA in spleen lymphocytes showed strong effects of both experimental factors. For lymphoproliferative response to ConA expressed as counts per minute (cpm), the two-way ANOVA revealed significant main effects of sex (F(1.28) = 8.02, *p* < 0.01) and genotype (F(1.28) = 182.12, *p* < 0.001), together with a significant sex × genotype interaction (F(1.28) = 22.51, *p* < 0.001). In fact, female and male 3xTg-AD mice had lower values of this immune parameter than observed in their corresponding NTg control animals (*p* < 0.001) (Figure 2A). A similar pattern was observed for the percentage response to ConA, with significant effects of sex (F(1.28) = 14.90, *p* < 0.001) and genotype (F(1.28) = 60.52, *p* < 0.001), and a significant interaction (F(1.28) = 11.82, *p* < 0.01). Both female and male 3xTg-AD had lower percentages of lymphoproliferation in response to ConA than their corresponding controls (Figure 2B) (*p* < 0.001). IL-2 production in response to ConA in the spleen also differed by sex (F(1.28) = 13.50, *p* < 0.001) and genotype (F(1.28) = 8.27, *p* < 0.01), although the interaction was not significant (F(1.28) = 0.03, *p* = 0.8742). However, post hoc analyses showed no statistical differences between experimental groups (Figure 2C). For thymus leukocytes, lymphocyte proliferation by ConA stimulation (cpm) showed significant effects of both experimental factors. The two-way ANOVA revealed a strong main effect of sex (F(1.28) = 29.83, *p*< 0.001) and a significant effect of genotype (F(1.28) = 17.14, *p* < 0.001), accompanied by a significant sex × genotype interaction (F(1.28) = 9.08, *p* < 0.01). When proliferation was expressed as a percentage, only the genotype factor reached significance (F(1.28) = 8.96, *p* < 0.01), whereas neither sex (F(1.28) = 0.01, *p* = 0.9349) nor the interaction (F(1.28) = 0.40, *p* = 0.5327) showed significant effects. Accordingly, female 3xTg-AD mice had lower lymphoproliferative response to ConA (both absolute counts and percentage) (Figure 2D,E) compared to female NTg animals (*p* < 0.01 and *p* < 0.001, respectively). IL-2 production in response to ConA stimulation in the thymus also demonstrated a clear genotype effect (F(1.28) = 20.48, *p* < 0.001). The sex × genotype interaction was significant (F(1.28) = 11.52, *p* < 0.01), while the main effect of sex was not (F(1.28) = 0.00, *p* = 1). Male 3xTg-AD mice had higher IL-2 release compared to male NTg animals (*p* < 0.05) (Figure 2F).

For the spleen lymphoproliferative response to LPS (Table 1), the two-way ANOVA showed strong main effects of sex and genotype, as well as a significant interaction. Males exhibited higher proliferative activity than females (F(1.28) = 22.41, *p* < 0.001), and 3xTg-AD animals showed markedly reduced responses compared with NTg controls (F(1.28) = 54.63, *p* < 0.001). The sex × genotype interaction was also significant (F(1.28) = 18.92, *p* < 0.001). However, post hoc analyses revealed that only male 3xTg-AD animals had lower values both absolute counts (c.p.m.) compared to their corresponding NTg controls (*p* < 0.05 and *p* < 0.001, respectively). Similarly, when expressed as a percentage, spleen lymphoproliferation followed the same pattern (Table 1), with significant effects of sex (F(1.28) = 16.84, *p* < 0.001), genotype (F(1.28) = 41.52, *p* < 0.001), and their interaction (F(1.28) = 12.77, *p* < 0.01). In fact, female 3xTg-AD presented lower counts and percentages of this immune function than their female control counterparts (*p* < 0.05 and *p* < 0.01, respectively).

**Figure 3:**
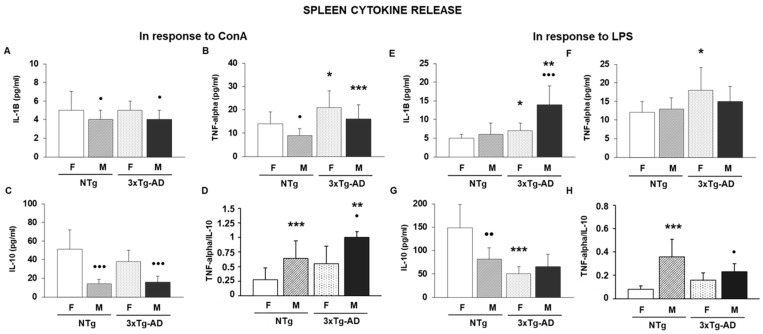
Proinflammatory (IL-1B, TNF-alpha), anti-inflammatory (IL-10) cytokine release evaluated in spleen cultures stimulated with ConA and LPS and TNF-alpha/IL-10 ratios from female and male non-transgenic (NTg) and triple-transgenic Alzheimer’s Disease (3xTg-AD) animals. (**A**) IL-1B, (**B**) TNF-alpha, (**C**) IL-10 levels (pg/mL) evaluated in spleen cultures stimulated with ConA. (**D**) TNF-alpha/IL-10 ratio of spleen cultures stimulated with ConA. (**E**) IL-1B, (**F**) TNF-alpha, (**G**) IL-10 levels (pg/mL) evaluated in spleen cultures stimulated with LPS. (**H**) TNF-alpha/IL-10 ratio of spleen cultures stimulated with LPS. * *p* < 0.05, ** *p* < 0.01 and *** *p* < 0.001 with respect to non-transgenic control; • *p* < 0.05, •• *p* < 0.01 and ••• *p* < 0.001 with respect to the values obtained in females of the same genetic condition.

Next, considering the impairments previously commented on in the lymphoproliferative response, we tried to decipher whether these alterations might be responsible for an altered cytokine response. For that, several classical pro-inflammatory (IL-1B and TNF-alpha) and anti-inflammatory (IL-10) cytokines were analyzed in the supernatants of spleen and thymus leukocyte cultures stimulated by ConA and LPS. Also, the TNF-alpha/IL-10 ratio, a good indicator of inflammation [60,61], was calculated. These data are illustrated in Figure 3 and Table 1.

Cytokine production following ConA stimulation showed distinct patterns depending on the immune mediator evaluated. IL-1B levels did not differ between groups, as the two-way ANOVA revealed no significant effects of sex (F(1.28) = 2.00, *p* = 0.168), genotype (F(1.28) = 0.00, *p* = 0.999), or their interaction (F(1.28) = 0.00, *p* = 0.999). For IL-10 production, this variable showed a strong influence of sex (F(1.28) = 52.63, *p* < 0.001), with females producing higher levels, and a moderate but significant effect of genotype (F(1.28) = 6.76, *p* < 0.05). The sex × genotype interaction did not reach significance (F(1.28) = 1.52, *p* = 0.227), suggesting that genotype affected IL-10 similarly in both sexes. Post hoc analyses revealed no statistical differences between Tg and NTg groups for both cytokines, IL-1B (Figure 3A) and IL-10 (Figure 3C). In contrast, TNF-alpha production exhibited significant main effects of both sex and genotype. Females showed higher TNF-alpha levels overall (F(1.28) = 20.57, *p* < 0.001), and genotype also increased TNF-alpha responses (F(1.28) = 11.14, *p* < 0.01). The interaction between both factors was not significant (F(1.28) = 0.00, *p* = 0.999), indicating additive rather than synergistic effects. In fact, the concentrations of TNF-alpha were higher in male and female 3xTg-AD animals compared to their corresponding NTg control mice (*p* < 0.05 and *p* < 0.001, respectively) (Figure 3B). The TNF-alpha/IL-10 ratio, used as an index of inflammatory balance, showed pronounced effects of both factors. Sex had a very strong impact (F(1.28) = 154.41, *p* < 0.001), and genotype also significantly increased the ratio (F(1.28) = 44.52, *p* < 0.001). Importantly, a significant sex × genotype interaction was detected (F(1.28) = 10.94, *p* < 0.01). Even so, only male 3xTg-AD animals presented higher values than the male NTg group (*p* < 0.01) (Figure 3D). In the case of cytokine production of thymus leukocytes stimulated by ConA, IL-1B levels exhibited a trend toward sex and interaction effects (both F(1.28) = 3.63, *p* = 0.066), while genotype produced a modest but significant increase (F(1.28) = 4.92, *p* < 0.05). Even so, post hoc analyses exhibited that male 3xTg-AD mice had higher levels of IL-1B compared to male NTg animals (*p* < 0.01) (Table 1). In contrast, TNF-alpha production showed robust effects of sex (F(1.28) = 18.67, *p* < 0.001) and genotype (F(1.28) = 12.47, *p* < 0.01), along with a significant interaction (F(1.28) = 4.89, *p* < 0.05). Among groups, both female and male 3xTg-AD mice had higher amounts of this cytokine than their corresponding NTg groups (*p* < 0.05) (Table 1). The TNF-alpha/IL-10 ratio showed very strong effects of sex (F(1.28) = 154.41, *p* < 0.001) and genotype (F(1.28) = 44.52, *p* < 0.001), together with a significant interaction (F(1.28) = 10.94, *p* < 0.01). Even so, only male 3xTg-AD presented higher values of TNF-alpha/IL-10 ratio compared to male NT mice (*p* < 0.01) (Table 1).

In the case of cytokines released from spleen leukocytes after LPS stimulation, significant main effects of sex (F(1.28) = 18.12, *p* < 0.001) and genotype (F(1.28) = 9.45, *p* < 0.01), and a significant interaction (F(1.28) = 7.62, *p* < 0.01) were obtained for IL-1B. In fact, female and male 3xTg-AD mice exhibited higher values of IL-1B compared to their NTg control animals (*p* < 0.05 and *p* < 0.01, respectively) (Figure 3E). For TNF-alpha, significant effects of sex (F(1.28) = 6.88, *p* < 0.05) and genotype (F(1.28) = 5.45, *p* < 0.05), although the interaction was not significant (F(1.28) = 2.01, *p* = 0.167). Only female 3xTg-AD mice had higher amounts of this cytokine than female NTg mice (*p* < 0.05) (Figure 3F). For IL-10 secretion, the two-way ANOVA reported a strong effect of sex (F(1.28) = 21.37, *p* < 0.001) and genotype (F(1.28) = 14.62, *p* < 0.001), and a significant interaction (F(1.28) = 5.98, *p* < 0.01). However, post hoc analyses revealed that female 3xTg-AD had lower values of IL-10 compared to the female NTg group (*p* < 0.001) (Figure 3G). Finally, with respect to the TNF-alpha/IL-10 ratios significant effects of sex (F(1.28) = 67.45, *p* < 0.001), genotype (F(1.28) = 24.83, *p* < 0.001), and a strong interaction (F(1.28) = 13.72, *p* < 0.001) were obtained. In fact, female 3xTg-AD had higher values compared to female NTg animals (*p* < 0.001) (Figure 3H). No signal was detected in the case of cytokines released in supernatant thymus leukocyte cultures stimulated by LPS.

**Figure 4:**
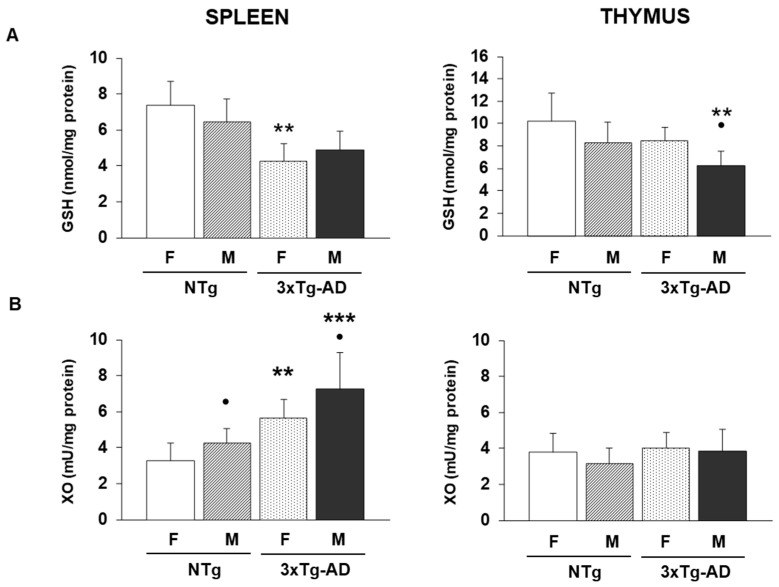
Oxidative stress parameters were analyzed in spleen and thymus leukocytes from female and male non-transgenic (NTg) and triple-transgenic Alzheimer’s Disease (3xTg-AD) animals. (**A-left**) Reduced glutathione (GSH) levels (nmol/mg protein) in spleen leukocytes (**A-right**) and in thymus leukocytes. (**B-left**) Xanthine oxidase (XO) activity (mU/mg protein) was evaluated in spleen leukocytes and (**B-right**). ** *p* < 0.01 and *** *p* < 0.001 with respect to non-transgenic control; • *p* < 0.05 with respect to the values obtained in females of the same genetic condition.

After that, we proposed to study whether these animals had an oxidative stress establishment since the close link between inflammation and oxidation exists [3,62]. For that, we analyzed the reduced glutathione (GSH) concentrations as well as the xanthine oxidase (XO) activity in spleen and thymus leukocytes from 3xTg-AD mice and their NTg counterparts. These data are in Figure 4.

For GSH concentrations evaluated in the spleen, the two-way ANOVA revealed a strong main effect of sex (F(1.28) = 54.12, *p* < 0.001) and a significant effect of genotype (F(1.28) = 98.44, *p* < 0.001). Also, a significant sex × genotype interaction (F(1.28) = 12.67, *p* = 0.0013) was obtained. Post hoc analyses revealed that female 3xTg-AD mice had lower GSH amounts compared to the female NTg group (*p* < 0.01) (Figure 4A). In the thymus, although GSH levels were also influenced by both factors (sex: (F(1.28) = 10.83, *p* < 0.01), and genotype: (F(1.28) = 15.92, *p* < 0.001) and the interaction approached significance (F(1.28) = 3.94, *p* < 0.05)), the statistical differenced observed in spleen was not exhibited in post hoc analyses female 3xTg-AD animals. Nonetheless, male 3xTg-AD animals presented lower GSH amounts compared to male NTg mice (*p* < 0.01).

Xanthine oxidase (XO) activity in the spleen showed robust effects of both sex and genotype (for sex: F(1.28) = 14.51, *p* < 0.001) and genotype: F(1.28) = 32.18, *p* < 0.001). A significant sex × genotype interaction was also observed (F(1.28) = 7.21, *p* < 0.05) (Figure 4B). In fact, female and male 3xTg-AD mice presented higher values compared to their corresponding NTg control animals (female and male NTg) (*p* < 0.01 and *p* < 0.001, respectively). This oxidant compound did not show any difference in the case of thymus leukocytes.

**Figure 5:**
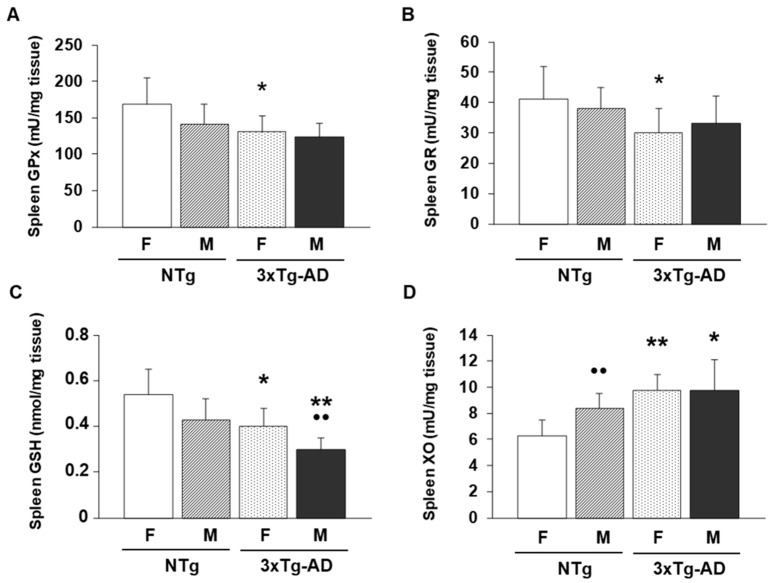
Oxidative stress parameters analyzed in spleen tissue from female and male non-transgenic (NTg) and triple-transgenic Alzheimer’s Disease (3xTg-AD) animals. (**A**) Glutathione peroxidase (GPx) activity (mU/mg tissue), (**B**) glutathione reductase (GR) activity (mU/mg tissue). (**C**) Reduced glutathione (GSH) levels (nmol/mg tissue). (**D**) Xanthine oxidase (XO) activity (mU/mg tissue). * *p* < 0.05 and ** *p* < 0.01 with respect to non-transgenic control; •• *p* < 0.01 with respect to the values obtained in females of the same genetic condition.

Finally, we wanted to determine whether these alterations, especially observed in the case of spleen leukocytes, could be maintained in the case of the complete organ. For that, we analyzed the previously cited antioxidant defense (GSH) and oxidant compounds (XO activity) together with the two enzymes, which form part of the glutathione cycle (glutathione Reductase-GR and glutathione Peroxidase-GPx). These data are illustrated in Figure 5.

GPx activity showed strong modulation by both sex and genotype. The two-way ANOVA revealed a significant main effect of sex (F(1.28) = 32.41, *p* < 0.001). Genotype also exerted a significant effect (F(1.28) = 84.12, *p* < 0.001). A significant sex × genotype interaction (F(1.28) = 10.56, *p* < 0.01) was observed. Similarly, GR activity was significantly influenced by both factors. Sex had a strong effect (F(1.28) = 14.83, *p* = 0.001). Genotype was also significantly significant (F(1.28) = 22.91, *p* < 0.001), but not the interaction F(1.28) = 3.92, *p* = 0.057). Post hoc analyses revealed that for GR and GPx activities, female 3xTg-AD mice presented lower values compared to female NTg animals (*p* < 0.05) (Figure 5A and Figure 5B, respectively).

For GSH concentrations, this parameter showed robust effects of sex and genotype (F(1.28) = 54.12, *p* < 0.001 and F(1.28) = 98.44, *p* < 0.001, respectively), as well as a significant interaction between factors (F(1.28) = 12.67, *p* < 0.01). Post hoc analyses showed a similar trend previously reported by GPx and GR, showing lower concentrations in female 3xTg-AD mice than female NTg animals (*p* < 0.05) (Figure 5C). Also, the male 3xTg-AD group had lower GSH amounts compared to their corresponding NTg controls (*p* < 0.01). Finally, XO activity showed strong modulation by both sex and genotype (F(1.28) = 14.51, *p* < 0.001 and F(1.28) = 32.18, *p* < 0.001). A significant sex × genotype interaction was also found (F(1.28) = 7.21, *p* < 0.05). In fact, XO activity (Figure 5D) was higher in female and male 3xTg-AD mice compared to female and male, respectively, NTg animals (*p* < 0.01 and *p* < 0.05, respectively).

### 3.1. Sex Differences

Several statistical sexual differences were observed. In fact, for the chemotaxis index evaluated in spleen leukocytes (Figure 1A), male NTg animals had lower values compared to female NTg animals (*p* < 0.001). This same trend was observed in the case of Tg groups, showing that male 3xTg-AD mice had lower chemotaxis indexes compared to female 3xTg-AD animals (*p* < 0.05). However, in the case of thymus leukocytes (Figure 1B), only male NTg animals had lower values in this immune function than female NTg mice (*p* < 0.05). For NK activity and in the specific case of spleen leukocytes (Figure 1C), male NTg and 3xTg-AD mice had lower percentages of lysis than their respective control groups (female NTg and 3xTg-AD mice) (*p* < 0.01). This same effect was exhibited in the case of the thymus (Figure 1D), showing a lower percentage of lysis in male NTg and 3xTg-AD mice compared to their corresponding controls (*p* < 0.05 and *p* < 0.001, respectively).

In the case of acquired immunity (Figure 2), while male NTg mice had higher values of spleen lymphoproliferative response to ConA than their controls (female NTg animals) (*p* < 0.001), male 3xTg-AD group only exhibits a significantly lower spleen lymphoproliferative response to ConA (evaluated as absolute counts) than female 3xTg-AD mice (*p* < 0.05). The IL-2 release was also lower in both male NTg and 3xTg-AD animals compared to female NTg (*p* < 0.05) and female 3xTg-AD mice (*p* < 0.01), respectively. For thymus leukocytes, male NTg animals had lower lymphoproliferative response to ConA (both absolute counts and percentage of stimulation) compared to female NTg mice (*p* < 0.01 and *p* < 0.001, respectively). This effect was also patent in the case of IL-2 release, observing a decrease in these cytokine concentrations compared to the female NTg group (*p* < 0.001). Indeed, male 3xTg-AD animals had lower IL-2 release than their corresponding controls (female 3xTg-AD animals) (*p* < 0.01).

For cytokines analyzed in supernatants of splenic leukocytes (Figure 3 and Table 1), male NTg and 3xTg-AD mice had lower IL-1B and IL-10 compared to their corresponding controls (female NTg and 3xTg-AD animals) (*p* < 0.05 for both groups in the case of IL-1B and *p* < 0.001 for both groups in the case of IL-10, respectively). However, for TNF-alpha, only the male NTg group had lower values compared to female NTg animals (*p* < 0.05), while in the case of TNF-alpha/IL-10 ratios, both male NTg and 3xTg-AD mice presented higher values than female NTg and 3xTg-AD groups (*p* < 0.001 and *p* < 0.05, respectively). For cytokines released in supernatants of thymus leukocytes stimulated by ConA (Table 1), male 3xTg-AD mice had higher concentrations of IL-1B compared to female 3xTg-AD animals (*p* < 0.05). This same effect was observed in the case of TNF-alpha levels (*p* < 0.01). For IL-10 levels, only the male NTg group had lower values of this cytokine compared to female NTg animals (*p* < 0.01). Finally, with respect to TNF-alpha/IL-10 ratios, male NTg and 3xTg-AD mice had higher values of this ratio than their corresponding controls (female NTg and female 3xTg-AD groups) (*p* < 0.01 and *p* < 0.05, respectively). With respect to the cytokine release in spleen cultures stimulated with LPS (Figure 3), while male 3xTg-AD mice had higher IL-1B (*p* < 0.001) together with TNF-alpha/IL-10 ratio (*p* < 0.05) compared to the female 3xTg-AD group, for IL-10 levels, only male NTg animals had lower values compared to female NTg mice (*p* < 0.01). Finally, male 3xTg AD animals had lower values compared to female 3xTg AD mice (*p* < 0.05).

In the case of oxidative stress parameters evaluated in spleen and thymus leukocytes (Figure 4), male NTg animals had higher XO activities in spleen leukocytes compared to female NTg mice (*p* < 0.05). This same trend was also observed for this parameter in the 3xTg-AD group (*p* < 0.05). Also, in the case of thymus leukocytes, male 3xTg-AD animals presented lower values of GSH than female 3xTg-AD mice (*p* < 0.05). Finally, in the case of spleen tissue (Figure 5), male 3xTg-AD mice had lower GSH amounts compared to female 3xTg-AD animals (*p* < 0.01). Indeed, male NTg animals had higher XO activity compared to female NTg mice (*p* < 0.01).

### 3.2. Effect of Regular Voluntary Exercise

After deciphering the immune impairments previously indicated in 3xTg-AD mice, we were prompted to study whether a regular voluntary physical exercise protocol might be enough to improve these parameters. The results obtained in the case of immune function evaluated in spleen and thymus leukocytes are summarized in Table 2 and Table 3.

For innate immunity functions evaluated in spleen leukocytes (Table 2) and concretely for the chemotaxis index, this variable was strongly influenced by all three biological factors. The three-way ANOVA revealed significant main effects of genotype (F(1.56) = 89.44, *p* < 0.001), sex (F(1.56) = 152.11, *p* < 0.001), and exercise (F(1.56) = 28.55, *p* < 0.001). Also, two-way interactions were significant, including genotype × exercise (F(1.56) = 7.12, *p* < 0.01) and sex × exercise (F(1.56) = 5.88, *p* < 0.05). Importantly, the three-way interaction was also significant (F(1.56) = 4.33, *p* < 0.05). In fact, male NTg performed exercise (M Exercise) had higher values of this immune function compared to male NTg control animals (*p* < 0.01). This same trend was observed in the case of female and male 3xTg-AD exercise (F Exercise and M Exercise). In fact, these animals presented higher values of chemotaxis index compared to their corresponding controls (female and male 3Tg-AD control animals) (*p* < 0.001). However, in the case of NK activity, neither genotype × exercise (F = 1.55, *p* = 0.218) nor sex × exercise (F = 0.44, *p* = 0.51) reached significance, and the three-way interaction was not significant (F(1.56) = 0.12, *p* = 0.73). In fact, only male 3xTg-AD E mice (M Exercise) had higher percentages of lysis than their corresponding controls (M Control) (*p* < 0.05).

Regarding acquired immunity evaluated in this location and in the specific case of lymphoproliferative response to ConA, a strong main effect of exercise was reported (F(1.56) = 9.44, *p* < 0.01). Significant two-way interaction was observed for genotype × exercise (F(1.56) = 6.88, *p* < 0.05), while sex × exercise showed a trend (F(1.56) = 3.44, *p* = 0.068). The three-way interaction was also significant (F(1.56) = 5.22, *p* < 0.05), indicating that exercise improved proliferative responses most effectively in 3xTg-AD males, who showed partial recovery from their baseline deficit. In fact, female and male 3xTg-AD E mice had higher c.p.m. compared to female and male 3xTg-AD control animals (*p* < 0.01 and *p* < 0.05, respectively). However, percentage proliferation showed a significant main effect of exercise (F(1.56) = 11.22, *p* < 0.01). Two-way interaction was significant for genotype × exercise (F(1.56) = 4.12, *p* < 0.05). The sex × exercise interaction was not significant (F = 2.55, *p* = 0.116). The three-way interaction approached significance (F(1.56) = 3.88, *p* = 0.053). Post hoc analyses revealed that for the percentage of lymphoproliferation stimulated by ConA, male NTg E animals had higher values compared to male NTg Control mice (*p* < 0.01). This effect was also patent in female 3xTg-AD animals exposed to voluntary exercise. In fact, these mice had higher percentages of stimulation by ConA compared to female 3xTg Control animals (*p* < 0.001). LPS-induced proliferation (in c.p.m.) showed a strong main effect of exercise (F(1.56) = 7.44, *p* < 0.01). A trend of significance was observed for genotype × exercise (F(1.56) = 3.22, *p* = 0.078). The sex × exercise interaction was not significant (F = 1.44, *p* = 0.235), and the three-way interaction was also not significant (F(1.56) = 0.88, *p* = 0.35). Similarly, the percentage of proliferation showed a significant main effect of exercise (F(1.56) = 5.22, *p* = 0.05). A trend was also observed for genotype × exercise (F(1.56) = 2.88, *p* = 0.095). The sex × exercise interaction was not significant (F = 0.55, *p* = 0.46), and the three-way interaction was also not significant (F(1.56) = 0.33, *p* = 0.57). Post hoc analyses showed that only female 3Tg-AD E mice had higher percentages of stimulation compared to their corresponding controls (female 3Tg-AD Control mice) (*p* < 0.01).

With respect to immune functions evaluated in thymus leukocytes (Table 3), the three-way ANOVA showed strong main effects of exercise (F(1.56) = 14.22, *p* < 0.001). Significant two-way interactions were observed for genotype × exercise (F(1.56) = 5.44, *p* < 0.05), while the sex × exercise interaction approached significance (F(1.56) = 3.77, *p* = 0.057). The three-way interaction was significant (F(1.56) = 4.01, *p* < 0.05). Both male NTg and 3xTg-AD mice exposed to exercise (M Exercise) had higher chemotaxis indexes compared to their corresponding controls (male NTg and 3xTg-AD Control groups) (*p* < 0.05). In the case of NK activity, exercise had a modest effect (F(1.56) = 4.55, *p* < 0.05). Neither genotype × exercise (F = 1.22, *p* = 0.27) nor sex × exercise (F = 0.33, *p* = 0.56) reached significance, and the three-way interaction was not significant (F(1.56) = 0.44, *p* = 0.51). Only male 3xTg-AD E mice had higher values of this immune function than male 3xTg-AD control animals (*p* < 0.05). For acquired immunity, ConA-induced proliferation showed strong main effects of exercise (F(1.56) = 12.44, *p* < 0.001). A significant two-way interaction was found for genotype × exercise (F(1.56) = 6.22, *p* < 0.05), while sex × exercise showed a trend (F(1.56) = 3.55, *p* = 0.065). The three-way interaction was significant (F(1.56) = 4.66, *p* < 0.05). In fact, male NTg and 3xTg E animals had higher absolute c.p.m. of lymphoproliferation in response to ConA compared to male NTg and 3xTg-AD Control mice (*p* < 0.01). This effect was also patent in the case of female 3xTg-AD E group. In fact, these mice had higher c.p.m. in response to ConA compared to their corresponding controls (female 3xTg-AD Controls) (*p* < 0.001). Percentage of proliferation showed similar patterns, with significant main effects of exercise (F(1.56) = 9.11, *p* < 0.01). Two-way interaction was significant for genotype × exercise (F(1.56) = 4.22, *p* < 0.05). The sex × exercise interaction was not significant (F = 1.77, *p* = 0.19). The three-way interaction approached significance (F(1.56) = 3.66, *p* = 0.061). However, post hoc analyses for the percentage of lymphoproliferative response stimulated by ConA showed that only female and male 3xTg-AD E groups had higher values than female and male 3xTg-AD control animals (*p* < 0.001). For LPS stimulation, a strong main effect of exercise (F(1.56) = 8.44, *p* < 0.01) was found. A significant trend was observed for genotype × exercise (F(1.56) = 3.11, *p* = 0.083). The sex × exercise interaction was not significant (F (1.56) = 0.88, *p* = 0.35), and the three-way interaction was also not significant (F (1.56) = 0.55, *p* = 0.46). For the percentage of LPS-stimulated proliferation, a strong effect was found in the case of exercise (F(1.56) = 5.22, *p* < 0.05). Neither genotype × exercise (F(1.56) = 2.66, *p* = 0.108) nor sex × exercise interaction was significant (F = 0.44, *p* = 0.51), and the three-way interaction was also not significant (F(1.56) = 0.33, *p* = 0.57). Male NTg E mice had higher c.p.m. compared to their corresponding controls (male NTg Controls) (*p* < 0.01). Indeed, female and male 3x-Tg-AD E groups had higher c.p.m. and percentages of lymphoproliferation in response to LPS compared to their corresponding controls (female and male 3xTg-AD Control animals) (*p* < 0.01).

## 4. Discussion

The present work is the first in which the deteriorated peripheral immune function (both innate and acquired immunity), as well as the presence of oxidative-inflammatory stress in immunocompetent organs such as the spleen and thymus, has been reported in both male and female 3xTg-AD mice at the age of 9 months. At this point, these animals are in an early neuropathological state. Moreover, voluntary physical exercise seems to alleviate these immune impairments, both in spleen and thymus leukocytes from 3xTg-AD mice.

The results obtained show that at 9 months of age, 3xTg-AD mice have, in general, impaired innate immune functions such as chemotaxis and NK antitumor cytotoxicity of leukocytes from spleen and thymus than those of NTg animals. These differences are statistically significant in the spleen for chemotaxis and in the thymus for NK activity, which can be understood by attending to the different nature of leukocytes present in both immune organs. Thus, the spleen is a secondary lymphoid organ, whereas the thymus is a primary one, which contains mainly immature T-cell precursors (thymocytes) with different properties compared to mature lymphocytes found in the spleen. Accordingly, with respect to the values of chemotaxis index, these are lower in the thymus than in the spleen, since thymocytes are worse prepared to respond to chemoattractant formylated peptide. These lower values of chemotaxis in the thymus than in the spleen were also reported in other strains of mice, such as Swiss and BALB/c [1,63]. Therefore, it is plausible that, in this context, we can observe statistically significant differences between NTg and 3xTg-AD in the spleen but not in the thymus. Moreover, lower values of chemotaxis in spleen leukocytes from 3xTg-AD than in NTg mice were also reported previously in females [31]. Although we have observed that 15-month-old female 3xTg-AD mice showed higher spleen and thymus leukocyte chemotaxis than the corresponding NTg, this was attributed to possible compensatory mechanisms against the effect of tau protein, which is completely established at this age in 3xTg-AD mice [39]. Therefore, we could suppose that at 9 months, this compensatory mechanism has not started and only appreciates the lower chemotaxis typical of animals with accelerated aging [64]. With respect to NK activity, which is carried out by a heterogeneous population of innate lymphoid cell (ILC) family, is lower in the thymus than in the spleen, as occurs in other strains of mice [63]. The lack of statistically significant differences between 3xTg-AD and NTg in NK activity of female spleen cells agrees with the results of a previous study [31], and it is maintained at 15 months of age [39].

The lymphoproliferative response to mitogens, a representative function of adaptive immunity, was lower in 3xTg-AD mice than in the corresponding NTg. When a T cell mitogen was used, as is the case with ConA [65], these effects are shown in both sexes in the lymphocytes of the spleen and only in females in the thymus. These sex differences could be due to a lower proliferative response to this mitogen of NTg males of this strain at this age. Whether LPS, a mitogen for B cells [66], was employed, the lower proliferative response of 3xTg-AD was observed, again, only in the thymus from females and in both sexes in the case of spleen, but with a clearer difference in males. The results in the female spleen agree with those previously obtained [31]. The lymphoproliferative response was more affected in the spleen than in the thymus in 3xTg AD mice. In fact, in AD, the spleen-brain axis has been shown as a critical component of its pathogenesis [67]. The lower proliferative response in 3xTg-AD mice *versus* NTg animals observed at 9 months disappears at 15 months of age, at a younger age in females [39], confirming the differences in peripheral immune functions with the development of disease. It was curious that the concentrations of IL-2 released in the cultures employed to analyze lymphoproliferation were not related to the results obtained for this immune function, since it is known that IL-2 promotes the proliferation of activated lymphocytes in both the thymus and the spleen [68,69]. Our results indicated no significant differences in IL-2 concentrations comparing 3xTg-AD and NTg, except for males, who show higher values in 3xTg-AD with ConA in the thymus and lower values with LPS in the spleen, in comparison to NTg mice. Since it is known that IL-2 is fundamental in thymus function [70] and 3xTg-AD males show a worse immune function than females, IL-2 could represent a compensatory mechanism in this immune organ for males. However, this disappears at 15 months of age [39]. In general, the better immune response in female than in male 3xTg AD mice could be related to the higher longevity of the former [71,72].

Altogether, the results obtained in the functions analyzed in spleen and thymus leukocytes from 3xTg-AD mice showed an impairment of peripheral immunity in these animals, with respect to controls, confirming an accelerated immunosenescence of 3xTg-AD, since previous results showed that those immune parameters decrease with aging [38,73]. These results agree with a lot of others that have indicated peripheral changes in the immune functions in AD patients [74,75,76,77]. Since some studies reported the involvement of systemic immunity in AD patients in the progression of the illness [78], the previous results obtained in 3xTg-AD mice, which showed the premature impairment of the peripheral immune cell functions, together with the present data indicating accelerated immunosenescence, could corroborate that involvement of the immune system in the disease progression.

Regarding the profile of pro- and anti-inflammatory cytokines, our findings indicate that leukocytes from 3xTg-AD mice generally produce higher values of proinflammatory mediators—particularly IL-1B and, most notably, TNF-alpha—compared with NTg controls. Consistent with these observations, our previous work demonstrated that elevated TNF-alpha production persists in 3xTg-AD mice at 15 months of age [39]. These results align with clinical evidence showing increased IL-1B secretion by mononuclear cells from AD patients relative to healthy individuals [78], as well as with reports of heightened proinflammatory marker expression in 3xTg-AD mice [72,79]. Furthermore, leukocytes from female 3xTg-AD mice released lower amounts of IL-10 following LPS stimulation compared with NTg counterparts. This reduction parallels findings in peripheral blood mononuclear cells from AD patients exposed to β-amyloid, which exhibit diminished IL-10 production relative to age-matched controls [80]. These lower values of IL-10 release and higher levels of TNF-alpha were also observed in those blood cells of AD patients after incubation with LPS [76]. Given that TNF-alpha signaling can impair IL-10–mediated anti-inflammatory responses by disrupting STAT3 activation, the imbalance observed here likely contributes to an enhanced proinflammatory state. Taken together, these data reveal an increased inflammatory ratio in 3xTg-AD mice at nine months of age, a shift that may play a meaningful role in driving or amplifying neuroinflammatory processes during early disease progression.

The redox parameters examined in this study indicate that 3xTg-AD mice exhibit an early decline in antioxidant defenses, supporting the notion that systemic oxidative imbalance contributes to AD pathology [6,7,8]. Consistent with the central role of glutathione (GSH) as a major endogenous antioxidant [81,82], leukocytes from 3xTg-AD mice showed lower GSH concentrations compared with NTg controls, with sex-dependent patterns across spleen and thymus. Although these differences were not observed at 15 months of age [39], the significantly lower values detected at 9 months suggest that redox disturbances emerge during early disease stages. The stronger differences observed in whole spleen tissue, particularly in males, point to a substantial contribution of stromal cells to the antioxidant environment, in line with the recognized immunoregulatory functions of spleen stromal elements [83]. Enzymatic components of the glutathione cycle were similarly affected. Thus, GPx and GR activities were lower in the spleen of 3xTg-AD mice, with significant reductions in females, and comparable findings have been reported in older transgenic males [84]. These alterations parallel those described in prematurely aging and old mice [1,40], as well as in experimental models of Alzheimer-like dementia induced by streptozotocin, which also show diminished GSH and associated enzyme activities [85]. Moreover, these lower concentrations of GSH were also found in blood mononuclear cells from AD patients [76]. Together, these observations reinforce the idea that impaired glutathione metabolism is a shared feature of aging and AD-related neurodegeneration. In contrast to the decline in antioxidant defenses, xanthine oxidase (XO) activity—an important source of ROS—was elevated in both spleen leukocytes and spleen tissue of 3xTg-AD mice. Increased XO activity has been implicated in multiple pathological conditions [86], including AD [87], and is consistently observed in prematurely and chronologically aged organisms [40,56]. The coexistence of lower antioxidant capacity and elevated oxidant production in 3xTg-AD mice therefore reflects a pronounced oxidative imbalance detected in peripheral immune organs.

Although immune-cell composition in the spleen varies by sex and mouse strain [88], the pattern of immune and redox alterations observed in 3xTg-AD mice closely resembles that described in other models of accelerated aging. Reduced chemotaxis, diminished NK activity, impaired lymphoproliferative responses, weakened antioxidant defenses, and increased XO activity have all been reported in prematurely and accelerated aging mice [56]. These parallels support the view that peripheral immunosenescence and oxi-inflammaging—processes strongly implicated in neurodegenerative diseases [89,90,91] and considered central to AD pathogenesis [92]—are already evident in 3xTg-AD mice at 9 months of age. Overall, the present findings indicate that early peripheral oxidative-inflammatory dysregulation may contribute to the progression of Alzheimer-related pathology. Given that immunosenescence and chronic oxidative stress are major drivers of accelerated aging and reduced longevity [1], targeting peripheral redox and immune alterations may represent a promising avenue for mitigating disease progression.

Sex-related differences in the neuroimmunoendocrine system, and in each of its constituent components, are well documented [37,38]. Although females of many mammalian species, including humans, generally exhibit greater longevity than males [37,38,93,94,95], and several age-associated diseases tend to appear earlier or progress more rapidly in men [96], other conditions—such as AD—show a higher prevalence or more severe expression in females [97]. In the present study, both male and female 3xTg-AD mice displayed impairments in immune function as well as alterations in redox and inflammatory state. However, males exhibited a slightly greater decline in adaptive immune responses, together with more pronounced oxidative and inflammatory profiles. This pattern is consistent with the accelerated aging phenotype frequently observed in male mice across strains, including both NTg and 3xTg-AD animals at various ages, and may contribute to the shorter lifespan typically reported for males [39].

Current evidence suggests that the periphery is playing a role that is not so well studied in AD. In this framework, one connection pathway could be the soluble Aβ, which can be found outside the brain since it crosses the BBB and enters the blood flow [98,99]. In the 3xTg-AD model, amyloid depositions across multiple organs, including the spleen, have been documented [84,100]. Thus, the antioxidant defenses’ decompensation observed in this organ could indicate that the Aβ accumulation as a source of oxidative stress and inflammation affects its functionality [10], supporting the relevance of peripheral alterations in the bidirectional cycle with the brain in early neuropathological stages of disease. In addition, recent findings highlight that peripheral immune pathways play a pivotal role in regulating brain Aβ and Tau homeostasis, particularly in response to physical exercise [101]. Although the positive effects of physical exercise interventions in 3xTg-AD mice on their behavioral and neuropathology were observed [55,102,103], on the peripheral immune system state, the results of the present study are the first showing its capacity to improve immune functions in these animals. In the immune functions studied, exercise manages to bring their values closer to those suitable for the strain and age of the animals. Thus, it had no effects on NTg females, but it did affect NTg males, who show impaired immunity compared to females, both due to deficiency (as occurs in chemotaxis index) or excess (in spleen lymphoproliferation). These effects are like those shown on immunity for other positive lifestyle strategies on immunosenescence [2]. Moreover, the modulatory role of peripheral immune response by exercise is very relevant in AD, since recent findings highlight that this is important for regulating brain Aβ and Tau homeostasis [101]. In fact, exercise has been proposed as a non-pharmacological intervention that targets peripheral immune-metabolic networks to mitigate AD pathology [101].

Several limitations should be acknowledged in this basic translational research study. The limited understanding of peripheral organ immunity in the context of AD constrains both the conceptual framework and the interpretation of the findings. Additional immune parameters and oxidative–inflammatory markers would have strengthened the analysis, particularly in the group of animals exposed to physical exercise. Moreover, assessing well-established AD biomarkers, such as Aβ40 and Aβ42 peptide levels or Aβ plaque burden, together with several well-defined behavioral alterations, without and under conditions of voluntary exercise, should be considered in future investigations. Through these studies, the neuropathological AD state and the possible beneficial effects of exercise could be clearly demonstrated. In fact, further studies will be needed to elucidate the mechanisms underlying the peripheral immune alterations reported here and their relationships with AD pathology.

## 5. Conclusions

In conclusion, the present findings in 3xTg-AD mice advance our understanding of AD by highlighting the contribution of peripheral immune alterations to early disease progression. Data demonstrate that both female and male 3xTg-AD mice exhibit, at 9 months of age, a generalized immune impairment affecting spleen and thymus leukocyte populations. These deficits emerge, presumably, during the initial stages of neuropathological development and appear amenable to modulation through voluntary physical exercise. Collectively, the results underscore the relevance of the peripheral immune system as a determinant of AD trajectory, supporting the use of peripheral immune markers to evaluate the efficacy of intervention strategies. Implementing beneficial lifestyle interventions such as voluntary exercise during this early window may be critical for mitigating or slowing the progression of Alzheimer-related pathology.

## Figures and Tables

**Table 1:** Lymphoproliferative response to LPS of spleen and thymus leukocytes, as well as thymus cytokine release in supernatants of cell cultures in response to ConA from female (F) and male (M) non-transgenic (NTg) and triple transgenic Alzheimer Disease (3xAD-Tg) mice.

	FNTg	MNTg	F3xTg-AD	M3Tg-AD
**Lymphoproliferative response to LPS**				
Spleen leukocytes				
c.p.m.	1331 ± 242	2676 ± 746 •••	995 ± 317	687 ± 217 *•••
%	264 ± 71	675 ± 243 ••	198 ± 56	193 ± 76 ***
Thymus leukocytes				
c.p.m.	883 ± 277	446 ± 106 ••	515 ± 134 **	419 ± 155
%	191 ± 44	180 ± 62	132 ± 40 *	157 ± 42
**Thymus cytokine amounts (pg/mL)**				
** In response to ConA**				
IL-1B	3 ± 0.4	4 ± 0.5	4 ± 0.5	6 ± 1 *•
TNF-alpha	4 ± 0.5	5.5 ± 1	6 ± 0.6 *	12 ± 2 *••
IL-10	9 ± 3	4 ± 1 ••	5 ± 1	5.5 ± 1
TNF-alpha /IL-10 ratio	0.44 ± 0.02	1.37 ± 0.5 ••	1.2 ± 0.6	2.18 ± 0.8 **•

Each value is the mean ± SD of 8 values corresponding to 8 animals, each value being the mean of duplicate assays. * *p* < 0.05, ** *p* < 0.01, *** *p* < 0.001 with respect to non-transgenic control (FNTg); • *p* < 0.05, •• *p* < 0.01, ••• *p* < 0.001 with respect to the values obtained in females of the same genetic condition.

**Table 2:** Effects of voluntary physical exercise (E) on spleen leukocytes from female (F) and male (M) non-transgenic (NTg) and triple transgenic Alzheimer Disease (3xTg-AD) mice.

	NTg				3xTg-AD			
	F Control	F Exercise	M Control	M Exercise	F Control	F Exercise	M Control	M Exercise
**Immune responses**								
Chemotaxis Index (C.I.)	1352 ± 290	1660 ± 613	715 ± 60 •••	955 ± 195 ††	703 ± 117 ***	1132 ± 215 †††	542 ± 105 ••	919 ± 195 †††
Natural Killer activity (%)	35 ± 12	42 ± 10	20 ± 6 ••	25 ± 7	26 ± 8	34 ± 8	15 ± 5 ••	21 ± 6 †
Lymphoproliferation								
Concanavaline A (c.p.m.)	2222 ± 562	2275 ± 547	3177 ± 302 •••	3111 ± 544	1119 ± 232 ***	2059 ± 633 ††	878 ± 218 **•	1155 ± 272 †
Concanavaline A (%)	441 ± 152	417 ± 38	804 ± 78 ••	514 ± 91 ††	225 ± 47 **	470 ± 70 †††	246 ± 71 ***	295 ± 53
LPS (c.p.m)	1331 ± 242	1441 ± 311	2676 ± 740 •••	3562 ± 1442	995 ± 317 *	1262 ± 304	687 ± 212 ***•	777 ± 132
LPS (%)	264 ± 71	262 ± 29	676 ± 243 ••	549 ± 91	198 ± 56	292 ± 42 ††	194 ± 76 ***	201 ± 41

Each value is the mean ± SD of eight values corresponding to eight animals, each value being the mean of duplicate assays. * *p* < 0.05, ** *p* < 0.01, *** *p* < 0.001 with respect to their corresponding NTg controls; • *p* < 0.05, •• *p* < 0.01, ••• *p* < 0.001 with respect to the values obtained in the same genetic condition of female animals; † *p* < 0.05, †† *p* < 0.01, ††† *p* < 0.001 with respect to the values obtained in animals with the same genetic condition, but not performing exercise.

**Table 3:** Effects of voluntary physical exercise (E) on thymus leukocytes from female (F) and male (M) non-transgenic (NTg) and triple transgenic Alzheimer Disease (3xTg-AD) mice.

	NTg				3xTg-AD			
	F Control	F Exercise	M Control	M Exercise	F Control	F Exercise	M Control	M Exercise
**Immune responses**								
Chemotaxis Index (C.I.)	1040 ± 242	1252 ± 310	746 ± 89 •	899 ± 145 †	850 ± 291	1049 ± 167	684 ± 110	867 ± 141 †•
Natural Killer activity (%)	28 ± 8	37 ± 11	19 ± 6•	20 ± 7	21 ± 5 *	27 ± 7	12 ± 3 •••	19 ± 6 †
Lymphoproliferation								
Concanavaline A (c.p.m.)	982 ± 55	1114 ± 280	501 ± 136 •••	769 ± 135 ††	576 ± 103 ***	917 ± 124 †††	437 ± 164	772 ± 241 ††
Concanavaline A (%)	215 ± 36	235 ± 38	204 ± 80	215 ± 34	148 ± 30 ***	237 ± 21 †††	161 ± 44	238 ± 25 †††
LPS (c.p.m)	883 ± 277	1002 ± 223	446 ± 106 ••	632 ± 118 ††	515 ± 134 **	762 ± 119 ††	419 ± 155	733 ± 180 ††
LPS (%)	191 ± 44	212 ± 30	180 ± 62	176 ± 27	132 ± 40 *	196 ± 8 ††	157 ± 43	232 ± 41 ††

Each value is the mean ± SD of eight values corresponding to eight animals, each value being the mean of duplicate assays. * *p* < 0.05, ** *p* < 0.01, *** *p* < 0.001 with respect to their corresponding NTg controls; • *p* < 0.05, •• *p* < 0.01, ••• *p* < 0.001 with respect to the values obtained in the same genetic condition of female animals; † *p* < 0.05, †† *p* < 0.01, ††† *p* < 0.001 with respect to the values obtained in animals with the same genetic condition, but not performing exercise.

## Data Availability

The original contributions presented in this study are included in the article. Further inquiries can be directed to the corresponding author(s).
